# An Overview of the Successful Application of Vibrational Spectroscopy Techniques to Quantify Nutraceuticals in Fruits and Plants

**DOI:** 10.3390/foods11030315

**Published:** 2022-01-24

**Authors:** Daniel Cozzolino

**Affiliations:** Centre for Nutrition and Food Sciences, Queensland Alliance for Agriculture and Food Innovation (QAAFI), The University of Queensland, Brisbane, QLD 4072, Australia; d.cozzolino@uq.edu.au

**Keywords:** spectroscopy, infrared, Raman, nutraceuticals, fruits, plants

## Abstract

Vibrational spectroscopy techniques are the most used techniques in the routine analysis of foods. This technique is widely utilised to measure and monitor the proximate chemical composition (e.g., protein, dry matter, fat and fibre) in an array of agricultural commodities, food ingredients and products. Developments in optics, instrumentation and hardware concomitantly with data analytics, have allowed for the progress in novel applications of these technologies in the field of nutraceutical and bio compound analysis. In recent years, several studies have demonstrated the capability of vibrational spectroscopy to evaluate and/or measure these nutraceuticals in a broad selection of fruit and plants as alternative to classical analytical approaches. This article highlights, as well as discusses, the challenges and opportunities that define the successful application of vibrational spectroscopy techniques, and the advantages that these techniques have to offer to evaluate and quantify nutraceuticals in fruits and plants.

## 1. Introduction

The nutritive value and health benefits of fruits and vegetables are considered high due to the occurrence of different chemical compounds and their functional properties (e.g., fibre) [1,2,3,4]. The nutrients and bioactive compounds found in different parts (e.g., leaves, fruits) of these plants include, in addition to the major chemical nutrients such as carbohydrate, protein, fibre, bioactive compounds such as phenolic and flavonoids, that are critical to the functionality of the human body [1,2,3,4]. Furthermore, minerals, vitamins, bioactive compounds (e.g., the so-called phytochemicals), as well as dietary fibre, contribute to preventing chronic diseases (e.g., cancer, cardiovascular disease, obesity, neurodegenerative disorder, type 2 diabetes mellitus and age-related disorders) [1,2,3,4]. Consequently, fruits and vegetables are considered functional foods due to their protective impact against most of the diseases described above [1,2,3,4]. Bioactive compounds, such as phytochemicals and vitamins (e.g., vitamin E, pro-vitamin A and vitamin C), can also be grouped as antioxidants, as they play a significant role in human health and nutrition [2,3,4]. For example, phenolics are part of the family of plant bioactive compounds, where flavonoids play several important roles in cancer and CVD prevention [1,2,3,4]. These compounds are considered to have a more powerful antioxidant activity than vitamins and carotenoids [1,2,3,4].

Antioxidants are implicated in the first line of defence mechanism against oxidative stress via the quenching of singlet oxygen in the human body [1,2,3,4]. Furthermore, frequent consumption of fruits and vegetables (e.g., phytonutrients) may possibly prevent cancer, cardiovascular diseases, diabetes, osteoporosis and age-related disorders, such as dementia [1,2,3,4]. Antioxidants have the capacity to scavenge free radicals, such as reactive oxygen species (ROS) and reactive nitrogen species (RNS), protecting the living cells against oxidative damages that may possibly lead to the onset of degenerative diseases [1,2]. These compounds also play a part in defining other quality attributes in both fruits and vegetables, such as colour and appearance [2,3,4]. It is well known that green colour is associated with the presence of pigments, such as chlorophyll, as well other phytochemicals, such as glucosinolates, while anthocyanins contribute to the range of red, orange, blue and purple colours [2,3,4]. Carotenoids are responsible for yellow, orange and red colours found in fruits and vegetables [2,3,4].

During routine analysis or quality control of fruit and vegetables, most of these phytochemicals are measured and quantified using an extensive variety of analytical methods and techniques that include high-performance liquid chromatography (HPLC), liquid chromatography (LC) and mass spectrometry (MS), as well as vibrational spectroscopy (e.g., near- and mid-infrared, Raman, UV, visible spectroscopy) [5,6,7,8,9,10,11,12]. The recent advances in instrumentation (e.g., LED, portable spectrophotometers) as well as in both software and data analytics (e.g., new algorithms, machine learning) have allowed for the expansion of novel and unique developments in a broad range of fields including chromatography [e.g., gas chromatography (G-C)], HPLC, MS and electrophoresis [5,6,7,8,9,10,11,12].

The last three decades have seen the growth of effective and rapid analytical systems that have integrated different data analytics (e.g., chemometrics, machine learning) with both instrumental techniques that have been utilised to evaluate the proximate composition, as well as the functional characteristics, of fruits, plant food ingredients, agricultural commodities and products [13,14,15]. These techniques are highly precise, targeting specific molecules and compounds, and boost the capability of handling several samples during routine and quality control, which expands the type and number of measurements, as well as the determination of several chemical compounds and nutrients in both research and industry laboratories [5,6,7,8,9,10,11]. While existing routine analytical instrumentation and technologies are used to efficiently measure several chemical compounds and/or nutrients in low concentration with high precision in a wide variety and types of samples, several of these methods and techniques (e.g., G-C, HPLC) usually involve or require a number of pre-processing stages prior to or during the analysis (e.g., isolation, filtration, extraction) [5,6,7,8,9,10,11,12,13]. On the other hand, less attention has been dedicated to the benefits and abilities of rapid instrumental methods based on vibrational spectroscopy, such as mid (MIR) and near (NIR) infrared and Raman spectroscopies, to analyse bioactive compounds and nutraceuticals in a wide range of agricultural commodities, food ingredients and food products [5,6,7,8,9,10,11,12,13,14,15].

The rewards of incorporating data-analytic methods, such as machine learning, into modern and rapid analytical instrumental techniques, such as vibrational spectroscopy (e.g., NIR, Raman, MIR) and other sensing technologies, including electronic noses, tongues and biosensors, have encouraged the development of novel approaches that are able to measure and monitor the chemical composition, nutritional value and functional properties of natural products and food ingredients in a wide variety of samples [5,6,7,8,9,10,11]. A growing number of these applications has been directly associated with the main purpose of ensuring that both raw ingredients and commodities meet the mandatory minimal quality control standards required by both the market and consumers (e.g., quality control standards) [5,6,7,8,9,10,11]. Additionally, the utilisation of these technologies by the food and nutraceutical industries, has allowed the identification and monitoring of changes in the chemical compositions of samples at numerous steps of the supply and value chain (e.g., transport, storage and processing), as well as during production and processing of these types of samples [5,6,7,8,9,10,11]. Developments in instrumentation, such as the utilisation of portable and handheld instruments, easy to use sample accessories, such as attenuated total reflectance (ATR) cells together with data analytics, have allowed for the development of new applications of vibrational spectroscopy in the field of nutraceutical and bio compound analysis. Furthermore, several recent investigations have demonstrated the capability of vibrational spectroscopy to evaluate and/or measure these nutraceuticals in a wide range of fruit and plants as alternative to classical analytical approaches, including LC-MS, G-C and HPLC [5,6,7,8,9,10,11,12,13,14,15,16,17].

This article highlights, as well as discusses, the challenges and opportunities that define the successful application of vibrational spectroscopy techniques, and the advantages that these techniques have to offer to evaluate and quantify bioactive compounds and nutraceuticals in fruits and plants. Please note that few examples will be provided in this document to illustrate the ability of these techniques to quantify nutraceuticals, as this paper did not aim to exhaustively review the extensive number of applications and papers available.

## 2. A Brief Background in Vibrational Spectroscopy

Near and mid infrared and Raman spectroscopy are part of a family of techniques that belong to vibrational spectroscopy [18,19,20,21,22,23]. These techniques have been applied to measure and quantify a wide range of chemical compounds, biochemical and functional properties in different food matrices, as well as in agricultural commodities, including fruits and vegetables [18,19,20,21,22,23]. The vibrational spectrum behaves as the so called “fingerprint”, and has the potential to quantify, qualify, characterise and elucidate the intrinsic characteristic of any biological samples [18,19,20,21,22,23]. The main advantages and specific characteristics of these techniques are associated with the simple steps required to pre-process or prepare the sample prior to analysis (e.g., extraction, dilution, concentration drying), the non-destructive and rapid nature of the analysis itself (e.g., a few seconds needed to collect a single spectrum), and have been comprehensively used to analyse a broad range of biological samples, both qualitatively and quantitatively [18,19,20,21,22,23].

Near-infrared spectroscopy deals with the interaction of matter and light in the NIR region of the electromagnetic spectrum between 750 and 2500 nm [9,10]. In infrared spectroscopy it is well known that when the infrared light interacts with the molecules present in a sample, the bonds of these molecules vibrate at different frequencies depending on the type of bond (e.g., energy bonding) [9,10]. In the NIR region the C–H, N–H and O–H vibration bonds are the most prevalent, determining the shape of the spectra of a given sample. Consequently, the result is a spectrum characterised by the overlap of these bonds in broad bands [9,10]. Although, most of the applications NIR spectroscopy deals with involves the measurement and/or quantification of the proximate composition (e.g., protein, fat, dry matter, etc.) of a sample, the NIR spectrum also reflects the physical properties or characteristics of the sample [9,10]. This is a unique characteristic of NIR spectroscopy that differentiates this technique from other instrumental analytical techniques. Therefore, the NIR spectra of samples can provide information, not only about the chemical composition of the food but also about its functionality [9,10].

Mid infrared is a high-resolution analytical tool to identify chemical constituents and to elucidate structural compounds present in a sample matrix [18,19,20,21,22,23]. Similarly, to NIR, MIR spectroscopy offers a rapid and non-destructive method to fingerprint samples. Different to NIR spectroscopy, MIR spectroscopy might require some level of sample preparation and can be influenced by the sample’s moisture, however, it shows better specificity and reproducibility [18,19,20,21,22,23]. Most of these frequencies will be absorbed when infrared light goes through a sample of an organic compound; however, frequencies will be transmitted through the sample without any absorption occurring [18,19,20,21,22,23]. Infrared absorption is related to the vibrational changes that happen inside a molecule when it is exposed to infrared radiation [18,19,20,21,22,23]. Different molecular bonds (C–C, C=C, C–O, C=O, C-H, O–H and N–H) have diverse vibrational frequencies [18,19,20,21,22,23]. If these characteristic bonds are present in an organic molecule, they can be identified by detecting the characteristic, frequency-absorption band in the infrared spectrum [18,19,20,21,22,23]. Consequently, the choice of either NIR or MIR as analytical techniques depends on the purpose of the specific application.

Raman is often considered as a complementary technique to both NIR and MIR spectroscopy [19,22]. Raman spectroscopy generates intense bands of functional groups with large polarizability (C=Cl, C=C and C=N) in contrast to the strong absorbance bands of functional groups with strong polarisation (O–H and C=O) in the infrared region [19,22]. The main bands in the spectra are due to local vibrational modes that emerge strongly in the infrared spectra, while stretching modes appear intensively in Raman spectra [19,22]. Raman spectroscopy is based on inelastic scattering from the interaction of incident radiations with vibrating molecules, where the main issue encountered in the application of this technique is its low sensitivity due to weak Raman scattering [19,22]. Therefore, a wide range of techniques have been developed to overcome and improve Raman signals, such as stimulated Raman scattering, coherent anti-Stokes Raman scattering, resonance Raman spectroscopy and surface-enhanced Raman spectroscopy (SERS) [19,22].

The last 10 years has seen an increase in research that reports the ability of image techniques, such as hyperspectral and multispectral techniques, to analyse bioactive compounds and nutraceuticals in fruit and plants [23,24,25,26,27]. The hyperspectral imaging technique blends the main characteristics of vibrational spectroscopy and imaging technique into one system providing with both spatial and spectral information from the sample [23,24,25,26,27]. A hyperspectral image is constructed by a hypercube with a three-dimensional dataset consisting of one spectral and two spatial dimensions [23,24,25,26,27]. Hyperspectral reflectance imaging is often achieved by combining the visible and NIR ranges and has been widely applied to quantify different attributes, whereas Raman spectroscopy can also be integrated with imaging technology in the so-called Raman imaging systems [28]. Raman imaging is highly sensitive to changes in specific chemicals present at low concentrations, enabling the visualisation of chemical distribution in the samples where other techniques, such as THz imaging, might have the potential to be an alternative to the utilisation of X-rays [28].

An important benefit of the utilisation of vibrational spectroscopy is that it describes and profiles the so-called fingerprinting of a given sample (e.g., raw material or ingredient) or a single compound [5,6,10,13,21]. The application of these techniques has also allowed for the expansion and implementation of rapid- and high-throughput methods, minimising the need for sample preparation or chromatographic separation [5,6,10,13,21]. With no need for such analytical requirements, vibrational spectroscopy can be utilised and implemented in several steps during pre- and post-harvest, during processing and storage and even at the market site [5,6,10,13,21]. Furthermore, the development of applications based on the utilisation of vibrational spectroscopy has been made possible by the incorporation of data analytics, allowing for the analysis and interpretation of the data collected, as well as to develop predictive models or calibrations used to quantify chemical compounds [5,6,10,13,21].

## 3. Data Analytics, Algorithms and Calibration

The implementation of instrumental techniques and their utilisation in modern analytical systems requires the utilisation of both data mining and data-analytic tools that are needed to extract the most relevant information from data [14,15,16,17,29,30,31]. Myriad methods and techniques have been developed (e.g., algorithms, pattern recognition, modelling techniques) to fulfil the main tasks imposed by the utilisation of modern instrumentation. These methods and techniques are the basis of, so-called, artificial intelligence (AI) and machine learning (ML) tools [14,15,16,17,29,30,31]. The group of techniques associated with machine learning are related to the application of statistical methods used to identify patterns in a given dataset [14,15,16,17,29,30,31]. These analytical data methods have been developed by a blend of disciplines, such as algebra, mathematics and statistics, that are utilised to analyse and process big datasets [14,15,16,17,29,30,31]. Multivariate data analysis has been very useful for processing large datasets derived from large datasets originating from a diverse group of instrumental methods [14,15,29,30,31].

It is well known that the implementation of vibrational spectroscopy (e.g., NIR, MIR) into a robust analytical method requires the utilisation of computational and data-analytic tools [14,16,17,24]. These tools are needed to extract the relevant information from the data collected during the analysis (e.g., NIR, MIR, Raman), which is later utilised to develop mathematical models that can be used to better understand the system being analysed [14,16,17,24]. Most of these data-analytic methods are based on a wide range of algorithms and statistical techniques [e.g., multiple linear regression (MLR), partial least squares regression (PLS), principal component analysis (PCA)] [14,16,17,24].

Overall, for the routine uses of vibrational spectroscopy, specifically during NIR applications, the development of a calibration is required. The calibration describes the relationships between the spectra and the reference data, and it is expressed as a mathematical model [14,16,17,24]. The calibration is evaluated by its capability to predict new samples and how good it is in relation to the reference method used [14,16,17,24]. As described above, calibrations are often needed during the implementation of NIR spectroscopy, as this technique is a low-selective technique, and, unlike the MIR spectra, captures only the overtones and combination tones of vibrations derived from the functional bonds [14,16,17,24]. During the analysis of samples using NIR spectroscopy, overtones appeared as highly overlapped peaks and required extensive use of chemometrics to process and extract back the signal related to the measured property of interest [14,16,17,24]. Once the NIR spectrometer was calibrated by combining it with a reliable predictive model, it could be deployed for routine use but required extensive testing to confirm its predictive ability to monitor any changes associated with the failure of the sensor, light source, electronics, etc. [14,16,17,24].

## 4. Samples, Sampling and Error

As important as the type of instrument, state of the art of the technology, new algorithms sampling and the inherent characteristics of the sample are playing a significant role in defining the accomplishments of the application of vibrational spectroscopy [14,15]. Recent studies and development in the theory of sampling consider that several characteristics or properties define the success of a given application using vibrational spectroscopy and that they are associated with the setting of the experimental conditions, the sampling, and the inherent characteristics of the sample [14,15,32,33,34,35]. Both the sampling method and the experimental conditions utilized to develop the instrumental method are directly associated with the sampling protocol, the preparation and processing of the sample (e.g., drying, grinding, other physical effects that might influence the sample physical properties) [14,15,36]. The inherent characteristics of the sample are associated with the property to be measured (e.g., limit of detection, range in concentration, standard error of the laboratory, number of samples) and the physical properties of the sample [14,32,33,34,35]. Preparing, pre-processing (e.g., grinding, homogenisation) and selecting samples to be incorporated into the application are not trivial tasks, and are often overlooked [14,32,33,34,35]. During sampling, pre-processing and preparation, several inconsistencies or errors can be generated and added into the overall error of the method (e.g., multiplicative effects) that have a direct and negative consequence in the expected error of prediction [14,32,33,34,35]. For example, different pre-processing steps, such as drying and grinding of the sample, can contribute significantly to exacerbate the analytical error (e.g., scatter, noise) [14,37]. Such interactions between the perturbation and the observation can be observed in most of the applications of vibrational spectroscopy and they will define the success or not of such applications, adding to the overall systematic error of the application [14,32,33,34,35]. Of high importance is the knowledge of the error of the laboratory (e.g., standard error between replicates), as this parameter is often utilised to evaluate or judge the robustness and the predictive ability of the calibration model in any given application.

**Figure 1:**
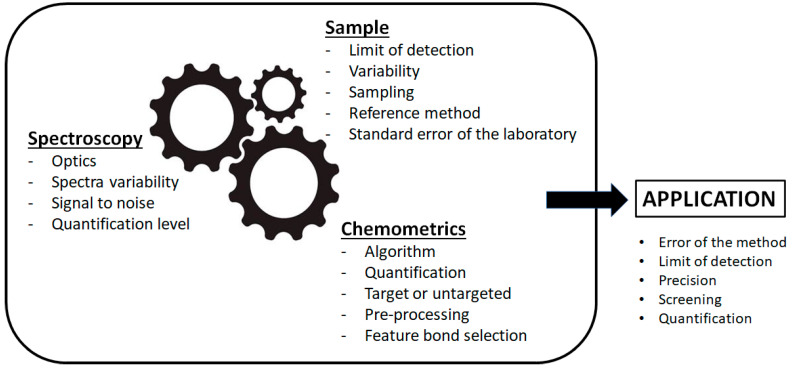
Schematic representation of how the spectroscopy, the sample and the chemometrics are interconnected to define the application.

An schematic representation of how the spectroscopy, the sample and the chemometrics are interconnected to define a given application in the field of vibrational spectroscopy is shown in Figure 1.

## 5. Examples of Applications of Vibrational Spectroscopy to Quantify Nutraceuticals

The ability of techniques, such as MIR or NIR spectroscopy, have been evaluated to quantify nutraceutical properties in different fruit samples [38]. Sinelli and collaborators used these techniques to predict total soluble solids (TSS), total phenols, total flavonoids, total anthocyanins and ascorbate in blueberries [39]. The reference data used to develop the calibrations were obtained by LC–MS and HPLC, where partial least squares (PLS) regression was used to develop the models between the reference and the spectral data [39]. Adequate calibration models (root mean standard error in cross-validation (RMSECV) = 0.50% and root mean standard error in test set validation (RMSEP) = 0.65%) and MIR regions (RMSECV = 0.30% and RMSEP = 0.36%) were reported to predict TSS. Total phenols (RMSECV = 0.14 mg catechin/g and RMSEP = 0.18 mg catechin/g), total flavonoids (RMSECV = 0.20 mg catechin/g and RMSEP = 0.25 mg catechin/g) and total anthocyanins (RMSECV = 0.25 mg malvidin/g and RMSEP = 0.22 mg catechin/g) were predicted using NIR and MIR spectroscopy [39]. The authors indicated that the predicted results developed for ascorbic acid, using either NIR or MIR spectroscopy, were not as good as those obtained for the prediction of phenolic parameters measured in the same sample dataset [39]. Early studies reported the ability of NIR spectroscopy to predict the presence of different bioactive compounds in tea, such as tea flavin and moisture content in samples of black tea [40]. Other authors demonstrated the ability of NIR spectroscopy to simultaneously predict the presence of alkaloids and phenolic compounds including epicatechin, epicatechin-3-gallate, epigallocatechin and epigallocatechin-3-gallate in leaves of green tea [40,41,42,43,44,45,46]. More recently, NIR spectroscopy was evaluated as a technique for the simultaneous quantification of the content of free amino acids, caffeine, total polyphenol content and amylose in green tea samples [44].

The ability of MIR spectroscopy to predict total antioxidant capacity and total phenolic content (TPC) in a diverse number of plant species was recently reported by researchers from Australia [47]. Calibration models were developed using standard normal variate (SNV) and smoothing as pre-processing applied to the MIR spectra [47]. This research showed high correlations between MIR spectroscopy and the reference data [47]. The R^2^ values reported by these authors were 0.96 for TPC, 0.83 for cupric reducing antioxidant potential (CUPRAC) and 0.911 for ferric reducing antioxidant potential (FRAP) [47]. The relative RMSE reported in the validation set showed that TPC could be predicted with higher accuracy than CUPRAC or FRAP [47]. It was highlighted by these authors that MIR spectroscopy could be considered as a promising technology to be utilised by plant breeders as well as in a variety of industries where rapid screening of numerous samples for antioxidant and/or phenolic content is required [47]. A recent review on the levels of beneficial anthocyanins and other antioxidative compounds in commercial, Australian-grown plums, and their measurement using different vibrational spectroscopy techniques, was published by the same authors [48]. In this study, the researchers evaluated a wide range of commercial plum varieties sourced from the central region of Queensland (e.g., grocery stores), including varieties developed and grown exclusively in Australia namely (e.g., Croc Egg and King Midas), as well as other commercial, well-known varieties such as black, Dapple Dandy, red and sugar plums [48]. The plum samples were analysed for antioxidants, phenolics, anthocyanins and mineral content, as well as using the ATR MIR instrument [48]. The reference and MIR spectral data were analysed using principal component analysis (PCA) [48]. The authors of this study concluded that commercially available Australian plums have comparable levels of health-promoting bioactive compounds to those reported internationally, although the amount of these bioactive compounds was highly dependent on the specific variety considered [48].

The utilisation of both NIR and MIR spectroscopy was also evaluated to predict antioxidant properties of red cabbage extract (*Brassica oleracea*) [49]. In this study ethanolic extracts of red cabbage were used to develop PLS calibrations based on vibrational spectroscopy [49]. This research was able to predict total and monomeric anthocyanins, total polyphenols and antioxidant capacity by ABTS (2,2-azino-bis(3-ethyl-benzothiazoline-6-sulfonate)) and DPPH (2,2-diphenyl-1- picrylhydrazyl). The calibration models reported by the authors demonstrated an excellent predictive capability of the method proposed (R^2^ > 0.98) [49].

The capability of vibrational spectroscopy techniques such as NIR and MIR spectroscopy, was tested to predict nutritional and antinutritional parameters in common beans (*Phaseolus vulgaris* L.) [50]. PLS regression models were developed for the quantification of protein, lipids, tannins, phytic acid and amino acids [50,51]. During calibration development, the use of pre-processing methods such as derivatives were tested by the researchers [50,51]. Overall, both techniques reported reliable methods to evaluate the proximate and antinutritional composition of bean flours [50,51]. The same researchers also reported the use of both NIR and MIR spectroscopy to quantify the phytochemical composition and in vitro antioxidant activity in several cultivars of *Phaseolus vulgaris* L. [51]. These authors reported that the best models were obtained by combining PLS regression with MIR spectroscopy (R^2^ calibration 0.86–0.99 and R^2^ validation 0.75–0.94), while for NIR the use of the first derivative of the spectra after normalisation provided the best models (R^2^ calibration 0.94–0.99 and R^2^ validation 0.85–0.97) [51]. The authors highlighted that it was possible to quantify single phenolic compounds in concentrations as low as ~5 μg g^−1^ and an in vitro antioxidant capacity of 2.1 μmol g^−1^ dw [51]. The utilisation of NIR spectroscopy combined with synergy interval partial least squares coupled competitive adaptive reweighted sampling (Si-CARS-PLS) and PCA analysis was reported to classify and predict walnut antioxidant properties [52]. The calibration statistics using Si-CARS-PLS were R^2^ = 0.96, RPD = 3.8 for TPC, R^2^ = 0.96, RPD = 3.3 for TFC, R^2^ = 0.96, RPD = 2.7 for the ABTS assay and R^2^ = 0.91, RPD = 2.6 for the FRAP assay [52].

During the last 10 years, several reviews and scientific papers have reported and highlighted that different vibrational spectroscopy techniques have the ability to monitor and quantify bio compounds, phytochemical and nutraceuticals in fruits and plant ingredients and materials [53,54,55,56,57,58,59,60,61,62,63]. We direct the reader’s attention to these papers and suggest the readers search for those applications in the vast amount of literature available.

## 6. Final Considerations and Conclusions

Techniques that belong to the family of vibrational spectroscopy (e.g., NIR, MIR, Raman) have been utilised during the routine analysis of samples by the food industry. The advantages of these techniques are associated with their ability to determine several chemical parameters of importance in foods in a single analysis. Many investigations have proven the capability of these techniques to evaluate, predict or measure functional properties in natural and nutraceutical ingredients, as well as products. This capability is associated with a well-know, but not generally explored, property of some of the vibrational spectroscopy techniques (e.g., NIR) that not only measure or quantify the chemical composition of a sample, but also collect the physical properties or functional characteristics of the sample. The prediction or quantification of nutraceuticals by vibrational spectroscopy is beyond the sole measurement or quantification of a given parameter. The analysis and interpretation of the spectrum provides a superior level of evidence about the sample that can be used to reveal not only composition, but also other properties alongside the interactions between components in the sample matrix.

The utilisation of these techniques generates large amounts of data. These data can be used to develop and implement advanced and rapid monitoring systems throughout the production and distribution of nutraceutical products. However, an important aspect to consider is the slow push and translation of these techniques across the whole bioactive and nutraceutical industries. Current innovations in instrumentation and data analytics have contributed to enhancing the current analytical capabilities, allowing the incorporation of these technologies directly into the processing of raw commodities and food ingredients. It has been demonstrated that the real-time monitoring of processes and storage conditions during food production is possible and is a reality in some cases. The different vibrational techniques available offer a wide range of technical resources to improve and incorporate decisions and management tools into the process and storage of nutraceuticals. In addition to the establishment of wireless sensor networks, these novel technical resources will result in an increase in the capability of vibrational spectroscopy, which can be utilised during the evaluation and monitoring of natural ingredients and products.

Despite the huge potential of vibrational spectroscopy as an analytical method, the use of these so-called emergent technologies to meet online inspection and quality prerequisites imposed by the industry will require further developments in R&D. The current global demands for fully nutritious, sustainable and safe foods are on top of the agenda for the modern food industry and, more importantly, the consumer. Regrettably, these questions are hampered by several overarching issues, including rising complexity in the supply chains, the effect of climate change (e.g., composition of raw materials and ingredients), growing and ageing population, security issues (e.g., contamination, fraud, traceability, origin, waste) and the continuous changes in consumers’ patterns or choices for nutritious and safe foods. As highlighted in this review, the development of applications of vibrational spectroscopy often requires the creation of calibration models that must include most of the expected variability, including different environmental conditions, samples sourced from different and diverse origins, appropriate spectral pre-processing, etc. However, the importance of this critical step is somehow ignored or underestimated.

The integration and utilisation of vibrational spectroscopy as a technique during the routine analysis of nutraceuticals has allowed for a surge in our ability to acquire data in a quick and useful manner, and, therefore, the capability of using such information in a more comprehensive way. We can expect that these techniques will be used to monitor the whole supply and value chain from the collection and harvesting of the ingredients throughout the processing, transport and storage of these goods. This new approach to the analysis of nutraceuticals will determine changes and innovations in the so-called management decision or expert systems utilised by the industry. Despite their enormous advantages, the application of vibrational spectroscopy as tool to monitor nutraceuticals during the process is lacking.

However, users need to have a different approach to how they interpret the calibration models developed to predict nutraceuticals, as the interpretation of nutraceuticals will determine a different level of complexity. A summary of the challenges and opportunities of the application of vibrational spectroscopy to analyse nutraceutical is shown in Table 1. The user needs to move away from a single number that explains what has already happened, to a more dynamic system that will provide information about the changes or trends in the system as a function of its complexity. This new methodology will require the use of new tools (e.g., aquaphotonics) [64], as well as the acceptance of multidisciplinary approaches, in order to interpret the spectra and the calibrations obtained to predict and quantify nutraceuticals in different sample matrices.

## Figures and Tables

**Table 1:** Summary of the challenges and opportunities facing the development of applications based on vibrational spectroscopy.

	Comments
**Challenges**	To understand the sources of spectral and sample variability prior to calibration development and implementation. To know the limit of detection and quantification of the reference method used, as well as the limit of quantification of vibrational spectroscopy. To define and interpret the error of the method considering the different sources, such as sampling, sample processing and preparation, reference method, instrumental technique. To collect a sample spectrum rather than collecting replicate spectra from the same sample. To validate the models with independent samples, beyond the overuse of cross validation. A proper interpretation of the calibration models, not only reporting the coefficient of determination. The standard error (e.g., calibration, cross-validation, prediction), bias, slope, etc., must be used to report the ability and robustness of a calibration to predict new samples. Issues associated with the lack of training and education.
**Opportunities**	The development of novel, easy to implement and use, methods to quantify nutraceuticals. The ability to integrate different disciplines (e.g., a tool to foster interdisciplinary research). The development of tools to monitor and quantify nutraceuticals in the entire supply and value chains. Opportunities in relation to training and education, workshops, etc.

## Data Availability

Not applicable.
